# A Case of Neurosyphilis With Penicillin Failure

**DOI:** 10.7759/cureus.21456

**Published:** 2022-01-20

**Authors:** Isaac Alsallamin, Afnan Alsallamin, Shade Greene, Faris Hammad, Ameed Bawwab

**Affiliations:** 1 Internal Medicine, St. Vincent Charity Medical Center, Cleveland, USA

**Keywords:** encephalitis, csf rpr, fta-abs, treponema pallidum, reversible dementia, penicillin resistance, penicillin failure, neurosyphilis

## Abstract

Neurosyphilis is any involvement of the central nervous system (CNS) by *Treponema pallidum*. The CNS may be involved at any stage of infection.

A 54-year-old previously healthy African American male was hospitalized due to a two-year history of progressive cognitive decline. One year after symptoms began, he developed, over a four-month period, gait disturbance resulting in frequent falls, speech impairment, worsening memory loss, psychosis, and an inability to perform activities of daily living.

A diagnosis of neurosyphilis was established upon cerebrospinal fluid (CSF) positive results and new changes in his mental status. The CSF showed predominant lymphocytic pleocytosis (17), elevated protein (111), and IgG index (4.25). Other viral and bacterial panels were negative. Intravenous penicillin G, 24 million units daily for 14 days, was given. Two months later, the patient was transferred to the hospital for altered behavior and mental status changes from the cognitive baseline. The repeat CSF rapid plasma reagin (RPR) titer (1:4) was the same as at initial diagnosis, despite appropriate treatment. Brain MRI showed progressive volume loss in both temporal lobes, thalamus, and cerebellum, consistent with evolving encephalitis. Treatment with intravenous penicillin G, 24 million units, was repeated. The patient improved clinically.

Hence, in emerging cases of syphilis, this patient has been diagnosed with a neurosyphilis flare, unresponsive to the usual dose and duration of penicillin. We recommended a repeat CSF examination every six months and having a lower threshold for CSF examination for possible flare or resistance. Our case showed a failure to respond to the usual course of penicillin, requiring a second course of IV Penicillin G, although no resistance to penicillin has been reported.

## Introduction

Neurosyphilis is any involvement of the central nervous system (CNS) by *Treponema pallidum* [1-3]. Neurosyphilis is a unique diagnosis, as it is hard to diagnose. The inability to culture *T. pallidum* in vitro makes it a non-practical method of diagnosis. The diagnosis is made by clinical findings and cerebrospinal fluid (CSF) results. CSF abnormalities, including mononuclear pleocytosis, increased protein concentrations, or reactivity in the CSF Venereal Disease Research Laboratory (VDRL) test. Regardless of the host immune status, neurosyphilis CSF results are associated with high rapid plasma reagin (RPR), usually a titer of ≥1:32 [1-3].

The CNS may be involved at any stage of infection [2-4]. Transmission is usually trans-sexual, but other routes are also reported. Cases of syphilis have been on the rise since 2000, with the highest rate among men having sex with men [1,3]. In the absence of symptoms, latent syphilis is a positive serology [3-7]. Clinical neurosyphilis can take the form of paretic neurosyphilis, neuropsychiatric manifestations, or Tabes dorsalis [2,3,7].

Clinical neurosyphilis developed in 20% of the cases after 10 years of untreated infection [2-4]. Most cases of neurosyphilis are among HIV patients [2,3]. Latent syphilis in immunocompetence is very unlikely to develop into clinical neurosyphilis with normal CSF findings [2,3]. Asymptomatic neurosyphilis is the diagnosis of history, serology, and positive CSF findings, including mononuclear pleocytosis, increased protein concentrations, or reactivity in the CSF VDRL test [6-8].

The slandered treatment of neurosyphilis was with penicillin. Symptomatic and asymptomatic neurosyphilis were treated with aqueous penicillin for 10 to 14 days [1,2], either with IV aqueous crystalline penicillin (G 3-4 mU/Q4h) or IM aqueous procaine penicillin (G plus oral probenecid 2.4 mU). Neurosyphilis with existing parenchymal damage may only arrest disease progression. In patients with confirmed penicillin allergy, allergy desensitization is recommended [2,3]. The use of other antibiotics has not been sufficiently studied [2,3,7].

Our case highlights neurosyphilis relapse and is refractory to slandered penicillin treatment due to noncompliance or inadequate course. Our case is of high value to encourage physicians to closely follow up with their patients and to have a low threshold for re-examining CSF for the possibility of flare or failure to respond.

## Case presentation

A 54-year-old previously healthy African American male was hospitalized due to a two-year history of progressive cognitive decline. One year after symptoms emerged, the patient resigned from his job due to his inability to perform his duties. He subsequently developed, over a four-month period, gait disturbance resulting in frequent falls, speech impairment, worsening memory loss, psychosis, and an inability to perform activities of daily living.

On examination, gait was wide-based, hypertonia and hyperreflexia were observed. His speech was dysarthric and dysphonic, with mixed aphasia. Neuropsychological testing revealed severe dementia, attention deficit, aggression, and delusions. Brain MRI revealed mesial temporal and insular fluid-attenuated inversion recovery (FLAIR) changes. An EEG revealed subclinical seizures. Blood tests including hepatic, thyroid function, and B12 were normal. HIV antibodies were negative; however, RPR in serum (1:1) and CSF (1:4) was positive. The CSF showed predominant lymphocytic pleocytosis (17), elevated protein (111), and IgG index (4.25). Other viral and bacterial panels were negative. The autoimmune encephalitis panel was negative for anti-N-methyl-d-aspartate (anti-NMDA) and anti-glutamic acid decarboxylase (anti-GAD). A PET scan revealed no neoplasm (Table 1).

**Table 1:** CSF analysis

Appearance	Clear, colorless	
Protein	111	High
Gram stain	Negative	
Glucose CSF: serum	79: 138	
WBC	17	
Differentials	Lymphocytic pleocytosis	
RPR titer	1:4	High
VDRL	High positive	
IgG index	4.25	

A diagnosis of neurosyphilis was established. Intravenous penicillin G, 24 million units daily for 14 days, was given. Thereafter, intramuscular benzathine penicillin of 2.4 million units per week was injected for three weeks. Sodium valproate was also started, and the patient was discharged to a rehabilitation facility with significant improvement in symptoms.

Two months later, the patient was transferred to the hospital for altered behavior and mental status changes from his cognitive baseline. Physical examination revealed inattention, agitation, hallucinations, and new neurologic deficits including impaired left lower extremity sensation, dysdiadokinesia, and past pointing. The repeat CSF RPR titer (1:4) was the same as at initial diagnosis, despite appropriate treatment (Table 2).

**Table 2:** CSF repeated after two months

Appearance	Clear, colorless	
Protein	73	High
Gram stain	Negative	
WBC	3	
Differentials		
Neutrophils	1%	
Monocyte	16%	
Lymphocytes	83%	
RPR titer	1:4	High
VDRL	Positive	

**Figure 1:**
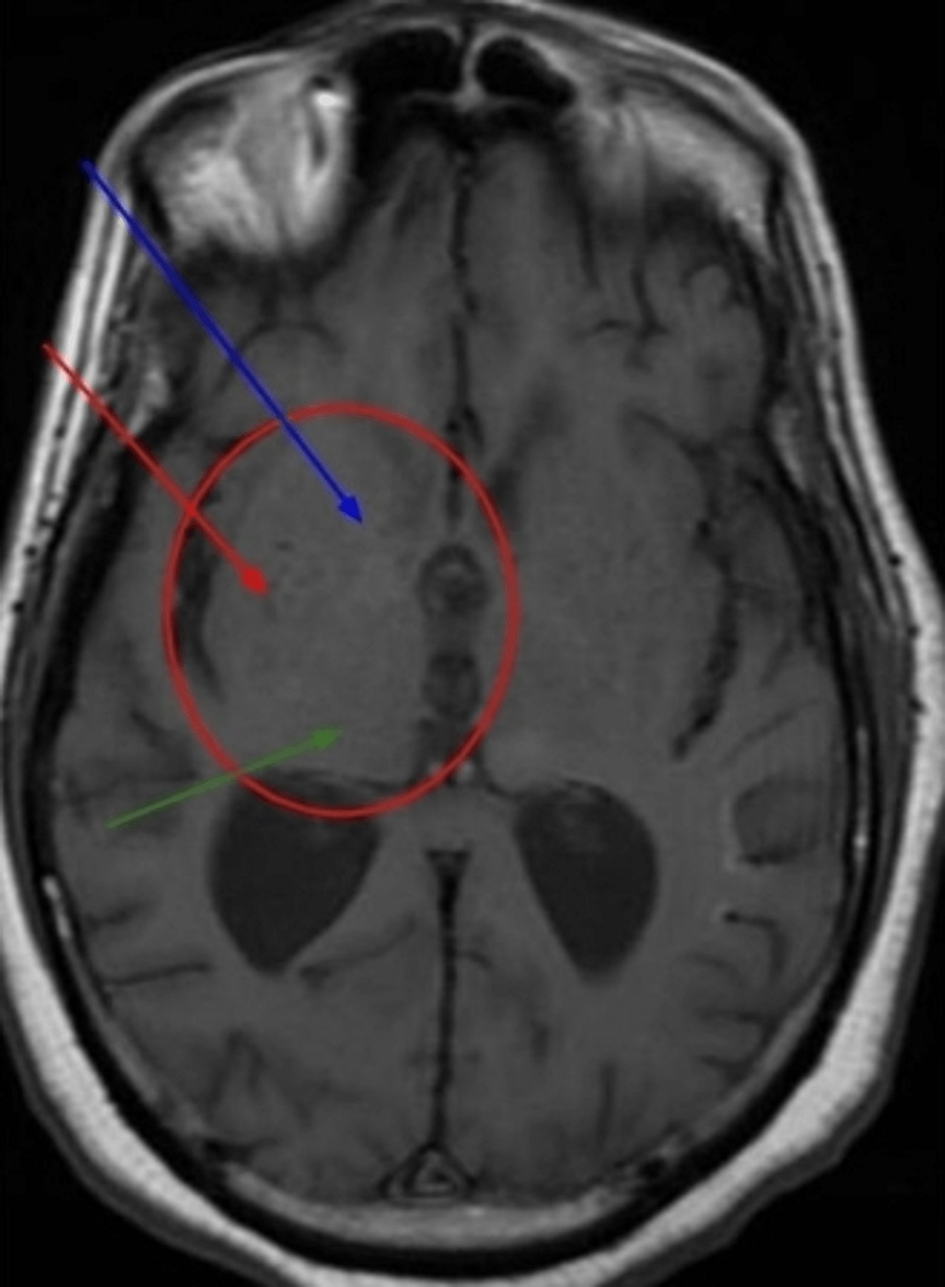
Brain MRI, T1 Bilateral basal ganglia atrophy (caudate, lentiform, and thalamus). Blue arrow: caudate, Red: lentiform, Green: thalamus, Circle: basal ganglia atrophy.

**Figure 2:**
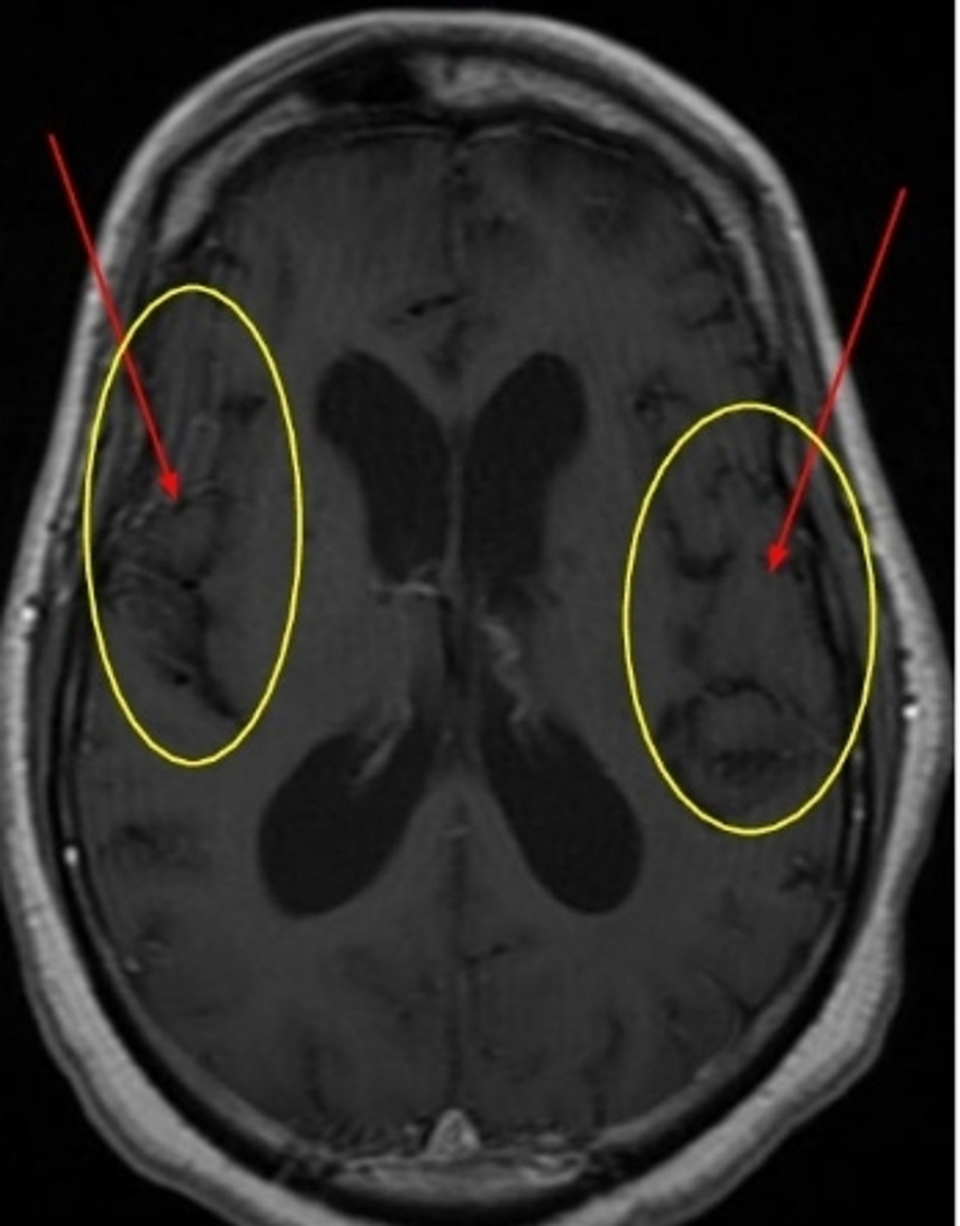
Brain MRI, T1 with gadolinium Bilateral temporal lobe atrophy. Circle: temporal lobe, Arrows: temporal lobe atrophy.

**Figure 3:**
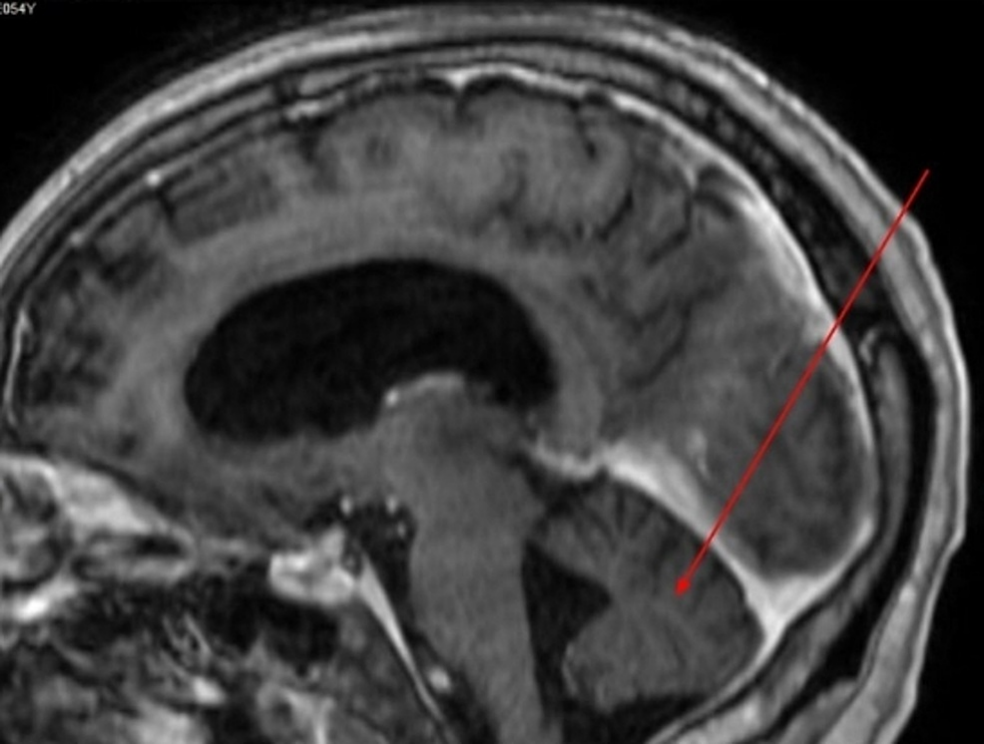
Brain MRI, multiplanar reconstructed sagittal Sagittal brain MRI, showing cerebellar atrophy (red arrow).

Brain MRI showed progressive volume loss in both temporal lobes, thalamus, and cerebellum consistent with evolving encephalitis (Figures 1-3).

The sodium valproate level was normal. Treatment with intravenous penicillin G (24 million units) was repeated. The patient improved clinically, became more oriented, and was able to perform his ADL with minimal assistance before his discharge to long-term acute care (LTAC) to complete the course of IV Penicillin. His sister was at his bedside talking about the significant improvement in his memory and orientation. A total of six weeks of follow-up since his diagnosis of the second flare, including hospitalization and LTAC length of stay. Unfortunately, he never showed up at our neurology clinic for follow-up.

## Discussion

The incidence of syphilis is increasing in the US. The standard of care is crystalloid penicillin G. Other antibiotics studied were less effective due to emerging resistance, although no resistance to penicillin has been reported [4,6,9-11]. When an antibiotic fails to kill the organism, this is called resistance. Treatment failure is different. It is not related to the antibiotic's ability to kill the organism. Many variables play a major role, such as patient compliance, duration of treatment, type, and dose of the antibiotics [6,10]. This patient was appropriately treated for neurosyphilis with 4 million units of intravenous crystalline penicillin G every 4 hours for 14 days and 2.4 million units intramuscularly once per week for three weeks [4-7]. Despite appropriate treatment, this immunocompetent patient clinically declined two months after treatment, and CSF re-evaluation indicated that retreatment of neurosyphilis was necessary due to persistently elevated RPR titer, protein, and lymphocytic pleocytosis. Hence, this patient has been diagnosed with neurosyphilis flair unresponsive to the usual dose and duration of penicillin. We recommended a repeat CSF examination every six months and having a lower threshold for CSF examination for possible flair or resistance.

The RPR and VDRL tests are recommended for screening and for quantitation of serum antibodies. The titer reflects disease activity, rising during the evolution of early syphilis, often exceeding 1:32 in secondary syphilis, and declining slowly thereafter without therapy. After treatment for early syphilis, a persistent fall of fourfold or more is considered an adequate response [4,6-8]. The CSF VDRL test is highly specific and, when reactive, is considered diagnostic of neurosyphilis [7,8]. A non-reactive FTA-ABS test on CSF may be used to rule out asymptomatic neurosyphilis [8]. All suspected patients with neurosyphilis must be completely evaluated for neurosyphilis with CSF analysis, RPR, and VDRL titer [2,3,7].

Penicillin G benzathine does not produce treponemicidal concentrations in CSF and should not be used for the treatment of neurosyphilis. Asymptomatic neurosyphilis may relapse as a symptomatic disease after treatment with benzathine penicillin, and the risk of relapse may be higher in HIV-infected patients. Our patient tested negative for HIV.

Patients with confirmed penicillin allergy must undergo desensitization and treatment with penicillin. Macrolide and doxycycline resistance have been reported [7,9,10]. One study involving genetic variants of *T. pallidum* bound to penicillin-binding protein showed four variant locations, but none of those variants was significant enough to alter *T. pallidum*'s response to penicillin [11].

There are limited studies on using ceftriaxone (1 g/d, given IM or IV for 8-10 days) to treat neurosyphilis, but a significant response to early syphilis was observed [12]. Our case represents a neurosyphilis relapse or flare rather than penicillin resistance, as a repeated course of IV Penicillin results in significant clinical improvement. There was no other antibiotic other than penicillin used, and no alternative diagnosis.

## Conclusions

Those patients who present with neurologic symptoms or neuropsychiatric deterioration with a history of treated or untreated neurosyphilis must be completely evaluated with brain imaging, CSF analysis, CSF RPR, and VDRL titer. High suspicion of neurosyphilis relapse or flare refractory to the usual penicillin treatment course, recurrence of neuropsychiatric symptoms, and signs after a history of treated neurosyphilis must be re-evaluated for treatment duration, proper antibiotics, and dose adjustment. Be aware that not all neurosyphilis cases respond to the usual course of penicillin. A six-month clinical follow-up period with RPR and VDRL titers is a proper follow-up protocol.
